# 

**Published:** 1916-04

**Authors:** 


### OUR NEW SUBSCRIBERS

Dr. R. H. Walker ------W. Va.
Dr T. G. Gantt ------------Ga.
Dr. W. H. Morgan --------Ala.
Dr. H. C. Martin --------W. Va.
Dr. M. L. Brown --------Texas
Dr. John A. Gibson --------Va.
Dr. R. M. Walker --------Texas
Dr. E. O. Powers -----------La.
Dr. W. W. Harloe ------W. Va.
Dr Chas. O. Hooks ------Texas
Dr. G. H. Fitzgerald ------Ariz.
Dr. Dallas Southard -----Texas
Dr. Francis Ridley --------W Va.
Dr. J. W. Kenney --------Texas
Dr. F. M. Wilson --------Texas
Dr E. L. Howard --------Texas
Dr. J. G. Rogers ----------S. C.
Dr. C. A. Dexter -----------Ga.
Dr. T. Adair ------------W. Va.
Dr. Vance Rawson --------Ky.
Dr. A. D. Simmington ------Ala.
Dr. W. B. Steavens ------W. Va.
Dr. J. L. McCarty ------W. Va.
Dr. J. T. Longest ----------Ark.
Dr. J. O. Matthews------Texas
Dr. J. C. Van Nuys ------Texas
Dr. J. A. Williams --------N. C.
Dr. L. F. Carlton ----------Fla.
Dr. H. G. Stoneham ------W. Va.
Dr. A. A. Williams --------Ga.
Dr. W. W. Coyner --------W. Va.
Dr. A. J. Buist ----------S. C.
Dr. L. O. Schwartz ------W. Va.
K. P. B. Bonner ----------N. C.
O. E. Clements ----------Texas
L. M. Ellis --------------Texas
H. H. Edmondson --------Tenn
Dr. G. C. Cole ------------Ga.
Dr. E. S. Colvin ----------Ga.
Dr. S. L. Katzoff ----------Ga.
Dr. H. W. Watson ----------Ga.
Dr. J. J. Martin ------------Ga.
Dr. J. Carothers ------------Ga.
Dr. O. H. Morris -----------Ga.
Dr. J. C. Oaksheets --------Ga.
Dr. H. M. Luning ----------Ga
Dr. L. M. Hill --------------Ga.
Dr. Thos. Slater ------------Ga.
Dr. Geo. S. Morse ----------Ga.
Dr. I. A. Dailey -----------Ga.

Dr. Geo. Carefoot ----------Ga.
Dr. D. B. Tallant ----------Ga.
Dr. O. T. Malone------------Ga.
Dr. H. J. Wright-----------Ga.
Dr. H. E. Duffy ------------Ga.
C. J. Nawrath ----------W. Va.
C. W. Hughes ------------Dela.
Jno. M. Williams ----------Ky.
P. G. Walker --------------Ky.
A. G. Kern --------------Tenn.
Timothy Griffith ----------Md.
Ira M. Hardy ------------N. C.
L. B. Auerbach ----------Texas
Wm. W. Tompkins ------W. Va.
W. P. Brogaw ------------Texas
L. G. Hair --------------N. C.
J. L. Austin -------------Texas
Jno. L. Lane --------------N. C.
J. H. Sanders ------------N. C.
Cecil Garronton -----------N. C.
Dr. M. W. McLarty --------Ga.
F. M. Griffin --------------S. C.
H. M. Stuckey -----------S. C.
T. D. Armistead -----------Va.
A. Herff-------------- --Texas
Conway & Allen ------------Ky.
E. V. Whitaker ------------La.
Saml Lindsey ------------S. C.
C. H. Waring ------------Miss.
J. H. Scott --------------Okla.
J. R. DeSteigner ---------Texas
A. L. Blesh --------------Okla.
Jno. H. Harden -----------S. C.
Anna M. Gove ------------N. C.
E. D. McKnight ----------Ark.
J. W. Knowlton ------------Ala.
Richard E. Vining ------W. Va.
F. J. Runyon ------------Tenn.
Chas. S. Holt ------------Ark.
C. O. Abernathy ----------N. C.
J. C. Watkins ------------Okla.
F. P. Sally ---------------S. C.
A. B. Nelson ------------W. Va.
A. F. Clark --------------Texas
Wm. A. Woody ----------Texas
E. S. Cross ---------------S. C.
F. B. Hardison ------------S. C.
Wm. D. Young -----------N. C.
J. W. Young ------------Texas
J. S. Maloy ------------W. Va.
Dr. M. R. Gibson ----------N. C.

Dr. J. G. Raby ____________N. C.
Dr. J. L. Duckett __________S. C.
Dr. D. A. Garrison ________N. C.
Dr. H. S. Keister ________W. Va.
Dr. V. F. Couch __________N. C.
Dr. Tilman Ramsey ________Ky.
Dr. T. C. Maguire __________Fla.
Dr. H. D. Wood ____________Ark.
Dr. Vance W. Brabham ____S. C.
Dr. L. Abrasom ____________La.
Dr. J. M. Crawford ______N. C.
Dr C. H. Maxwell ______W. Va.
Dr. O. P. Nuckols __________Ky.
Dr. George A. Griffith ____N. C.
Dr. C. I. Wall __________W. Va.
Dr. Thel. Hooks __________N. C.
Dr. Roger Coke ________Texas
Dr. M. C. Scott ____________Va.
Drs. Newton & Richer ____Texas
Dr. Robert Wilson ________S. C.
Dr. William L. Dunn ______N. C.
Dr. H. A. Barow __________S. C.
Dr. T. H. Marble __________Tenn
Dr. Robert Riston ______W. Va.
Dr. C. B. Backus ________W. Va.
Dr. J. E. Cannady ______W. Va.
Dr. W. S. Beane ________W. Va.
Dr. L. C. Stokes ________S. C.
Dr. A M. Showalter ________Va.
Dr. R. Moreland ____________Ky.
Dr. William H. Wadsworth N. C.

